# The Association between Obesity and Severity in Patients with Coronavirus Disease 2019: a Retrospective, Single-center Study, Wuhan

**DOI:** 10.7150/ijms.54655

**Published:** 2021-02-18

**Authors:** Jishou Zhang, Yao Xu, Bo Shen, Hua He, Mingxiao Liu, Mengmeng Zhao, Jianfang Liu, Shuwan Xu, Wei Pan, Jing Ye, Zhen Wang, Di Ye, Menglin Liu, Dan Li, Zhen Luo, Yongqi Feng, Menglong Wang, Jun Wan

**Affiliations:** 1 Department of Cardiology, Renmin Hospital of Wuhan University, Wuhan 430060, China.; 2 Cardiovascular Research Institute, Wuhan University, Wuhan, China.; 3 Hubei Key Laboratory of Cardiology, Wuhan, China.; 4 Department of Emergency, Renmin Hospital of Wuhan University, Wuhan, China.; 5 Department of Pediatrics, Renmin Hospital of Wuhan University, Wuhan, China.; 6 Medical Quality Management Office, Renmin Hospital of Wuhan University, Wuhan, China.; 7 Department of Medical Affaires, Renmin Hospital of Wuhan University, China.

**Keywords:** coronavirus disease 2019, body mass index, obesity, severity.

## Abstract

**Aim:** In other respiratory infectious diseases, obesity may be associated with a poor outcome. For coronavirus disease 2019 (COVID-19), the association between obesity and severity or prognosis requires further analysis.

**Methods:** This was a retrospective, single-center study. Hospitalized patients were recruited in Renmin Hospital of Wuhan University from January 2, 2020 to February 20, 2020. The data of body mass index (BMI) was obtained from follow-up of surviving patients. According to BMI, normal weight was defined as 18.5-23.9 kg/m^2^, overweight as 24.0-27.9 kg/m^2^ and obesity as > 28.0 kg/m^2^.

**Results:** A total of 463 patients were enrolled, of which 242 (52.3%) patients were in the normal weight group; 179 (38.7%) were in the overweight group; and 42 (9.1%) were in the obesity group. Compared to the normal group, obese patients were more likely to have a higher heart rate; lower finger oxygen saturation; higher levels of white blood cells, neutrophil counts, basophil counts, intravenous glucose, triacylglycerol, uric acid, alanine aminotransferase, creatine kinase-MB, CD19+ cell counts and percentage; and lower levels of monocyte percentage, high density lipoprotein and CD3+ cell percentage. In addition, the proportions of hypertension (21.5% vs. 42.6%) and severe+critical illness (47.8 vs. 81.0 %) were significantly higher in the obesity group than those in normal group. However, no significant differences were observed between the normal and obesity groups in critical illness, organ damage and defined endpoint (mechanical ventilation or intensive care unit). Multiple logistic regression showed that obesity increased the risk of developing severe+critical illness (Odd ratio 3.586, 95% CI 1.550-8.298, P=0.003) in patients with COVID-19, and did not affect the risk of critical illness, organ damage and endpoints. Overweight did not affect the risk of severity, organ damage or endpoint in patients with COVID-19.

**Conclusion:** Obesity may be a risk factor for developing severity in patients with COVID-19.

## Introduction

The coronavirus disease 2019 (COVID-19) pandemic, caused by severe acute respiratory syndrome coronavirus 2 (SARS-CoV-2), currently is posing a huge health risk to populations around the world. Many COVID-19 patients have underlying diseases, such as hypertension, diabetes, coronary artery disease, chronic obstructive pulmonary disease (COPD), and liver disease, which contribute to the severity and poor prognosis 1-3. In an observational study from China, the most common comorbidity was hypertension (16.9%), followed by diabetes (8.2%), COPD (1.5%) and chronic kidney diseases (21; 1.3%) 4. In another observational study from New York, USA, the most prevalent comorbidities were hypertension (56.6%), obesity (41.7%), diabetes (33.8%) and coronary artery disease (11.1%) 5.

Obesity is another global epidemic, especially in some western countries 6. Obesity is associated with increased morbidity of hypertension, other cardiovascular diseases, diabetes and cancers 6-8. Since hypertension and diabetes increase the risk of severe COVID-19 9,10, it is necessary to investigate the association between obesity and severe illness or mortality due to COVID-19. Obesity can increase the risk of hospitalization with pneumonia among males 11 but may be independently associated with lower short-term mortality in patients with pneumonia 12. In other infectious diseases, obesity may be associated with a poor outcome in pandemic H1N1 influenza infection 13. Therefore, we designed this study to analyze the associations among normal, overweight and obese patients in an enrolled COVID-19 cohort from Wuhan, China. The classification was based on body mass index (BMI). Since obesity rates were relatively low in China and other developing countries, our study may provide an aid to the treatment of obese patients with COVID-19 in these countries.

## Methods

### Study design and participants

This was a retrospective study. The study involving human participants was reviewed and approved by the Institutional Ethics Board of Renmin Hospital of Wuhan University.

**Figure 1:**
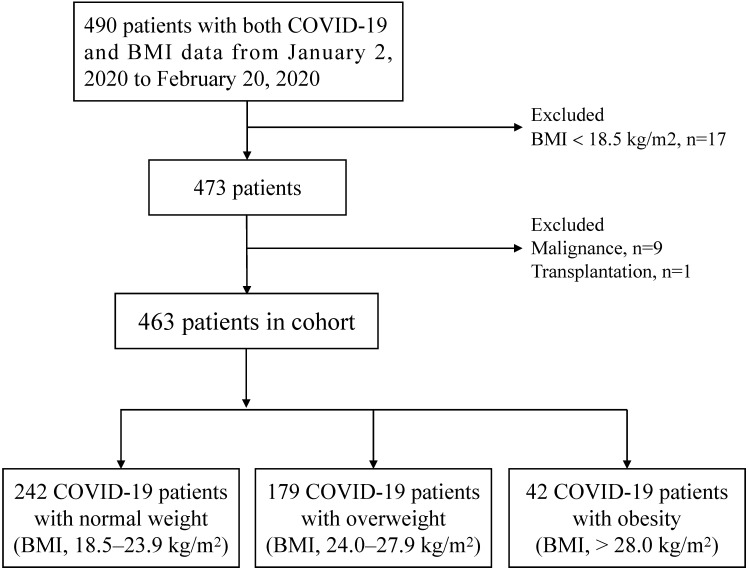
The flowchart showing the strategy of participant enrollment.

Consecutive COVID-19 in-hospital patients were recruited in Renmin Hospital of Wuhan University from January 2, 2020 to February 20, 2020. Renmin Hospital of Wuhan University was a designated hospital during epidemic in Wuhan, which mainly admitted non-mild patients with COVID-19. All patients with COVID-19 were diagnosed with fluorescence reverse transcriptase-polymerase chain reaction (fRT-PCR) assay for SARS-CoV-2 according to the Diagnosis and Treatment of Novel Coronavirus Pneumonia (6th edition) guidelines published by the National Health Commission of China 14. The data of BMI was obtained from the telephone follow-up of surviving patients and the follow-up time was two weeks after discharge. According to the Chinese-specific cut-offs for general adiposity, BMI 18.5-23.9 kg/m^2^ is defined as normal weight, BMI 24.0-27.9 kg/m^2^ as overweight and BMI > 28.0 kg/m^2^ as general obesity 15,16. Exclusion criteria: patients without BMI data or BMI < 18.5, pregnancy, acute myocardial infarction, malignancy and transplantation. A total of 490 COVID-19 patients had BMI data. After excluding 27 patients based on the exclusion criteria, a total of 463 patients, ultimately, were enrolled. Patients were divided into three groups: normal weight (n=242), overweight (n=179) and obesity (n=42). The strategy of participant enrollment was showed in **Figure 1**.

According to the Diagnosis and Treatment of Novel Coronavirus Pneumonia (6th edition) guidelines 14, patients were classified as mild/moderate, severe and critical severe. The detailed criteria of classification include: Mild: mild signs or symptoms, without signs of pneumonia; Moderate: general signs or symptoms, imaging shows pneumonia; Severe: Meeting any of the following conditions: 1. respiratory rate ≥30 beats per minute; 2. resting SpO2 ≤ 93%; 3. arterial PaO2/FiO2 ≤ 300 mmHg; Critical: Meeting any of the following conditions: 1. respiratory failure, needing mechanical ventilation; 2. shock; 3. combined with other organ failure, requiring intensive care. All patients were followed by doctors for 30 days during hospitalization, unless the patients were discharged.

### Data collection

The clinical data (e.g. basic information, initial symptoms, signs at admission, comorbidities, the first laboratory findings, treatment, complications and outcomes) were extracted and obtained by experienced clinicians based on the medical records system of the hospital. And, the first laboratory findings were obtained within three days of admission. Manifestations of lung on computed tomography (CT) were extracted and summarized by integrating the documentation or description in medical charts.

### Definition

Since BMI data (height and weight data) were obtained from the follow-up of survival patients, we defined the primary endpoint as a composite of mechanical ventilation or intensive care unit (ICU) within 30 days. The organ damage was a composite of shock, acute myocardial injury, acute kidney injury or acute liver injury within 30 days of hospitalization. Organ damages or injuries were defined as follows: Acute myocardial injury: Plasma hypersensitivity troponin I (hs-TnI) levels above the upper limit of normal value; Acute liver injury: Alanine aminotransferase (ALT) ≥150 U/L; Acute kidney injury: Patients satisfied either of the following conditions: 1) the highest serum creatinine level increased by more than 26.5 μmol/L (0.3 mg/dL) within 48 hours; or 2) Serum creatinine exceeded the baseline value by 1.5-fold (confirmed or estimated to occur within 7 days). Shock was considered based on the criteria: Hypotensive patients with failed volume resuscitation were administered vasopressors to maintain blood pressure.

### Statistics analysis

No assumptions were made regarding missing data. SPSS 22.0 software and GraphPad prism 7.0 were used to perform Statistical analysis. The continuous variables were expressed as the median - interquartile range (IQR) or mean ± standard error of the mean (SEM), and the differences between any 2 groups were performed by the Mann-Whitney test or student t' test. The categorical variables were presented as number (percentages) and were compared using chi-square tests, or fisher's exact test, if the data was limited. Logistic regression analysis was applied to investigate the association between overweight or obesity and severity, organ damage or endpoint, with the odd ratio (OR) and 95% confidence interval (95% CI) being reported. A two-tail p value < 0.05 was considered statistically significant.

## Results

### Basic characteristics of COVID-19 patients among the normal, overweight and obesity groups

A total of 463 patients diagnosed with COVID-19 were enrolled** (Figure 1)**, of which 242 patients (52.3%) were divided into the normal weight group, 179 patients (38.7%) into the overweight group and 42 (9.1%) into the obesity group. The median [IQR] ages were 62 (49, 68) years in the normal group, 59 (50, 67) in the overweight group and 63 (45, 68) in the obesity group, and no significant differences were observed among the three groups. With the advancement of BMI, there were no significant differences in the proportion of males (45.5% vs. 50.8% vs. 54.8%) among the three groups. Similarly, there were no significant differences in the proportions of several initial symptoms among the three groups, including fever, cough, fatigue, chest distress, anorexia, diarrhea and muscle ache. Regarding signs at admission, compared to the normal group, the obesity group had higher heart rate (median, 83 vs. 85 vs. 90 bpm/min) and lower finger oxygen saturation (median, 98 vs. 96 vs. 96 %). However, there were no significant differences among the three groups in respiratory rate and temperature **(Table 1).**

Regarding comorbidities, the proportion of COVID-19 patients with hypertension (21.5% vs. 31.3% vs. 42.6%) increased with the advancement of BMI. There were no significant differences among the three groups in other comorbidities (e.g., diabetes, coronary artery disease, arrhythmia, COPD/asthma, cerebrovascular disease, chronic liver disease and renal disease). Furthermore, the duration from disease onset to admission was also analyzed, and no differences were found among the three groups **(Table 1).**

### First findings of laboratory tests within three days of admission in patients with COVID-19

We first compared the blood routines among the three groups. Compared to the normal group, the overweight and obesity groups had higher levels of white blood cells (WBC) (median, 4.88 vs. 5.76 vs. 6.49 ×10^9^/L), neutrophils (median, 3.23 vs. 3.91 vs. 4.34 ×10^9^/L) and basophilic granulocytes (median, 0.01 vs. 0.01 vs. 0.02 ×10^9^/L). In addition, the levels of C-reactive protein (CRP) (median, 33.1 vs. 41.65 mg/L) and procalcitonin (median, 0.046 vs. 0.06 ng/mL) were also higher in the obesity group than those in the normal weight group, although the differences were not significant, possibly because the size of the obesity group was small. These results indicated that the patients in the obesity group had more severe secondary bacterial infections **(Table 2).**

Compared to the normal group, both the overweight and obesity groups had higher intravenous glucose (median, 5.33 vs. 5.76 vs. 6.29 mmol/L). Triacylglycerol (median, 1.14 vs. 1.34 mmol/L) and uric acid (median, 232 vs. 305 μmol/L) levels were higher and high-density lipoprotein levels (median, 0.97 vs. 0.88 mmol/L) were lower in the obesity group than those in the normal weight group. However, no significant differences were observed in low-density lipoprotein and cholesterol. These results may suggest that patients in the obesity group had worse metabolic disorders** (Table 2).**

With the advancement of BMI, the levels of alanine aminotransferase (median, 20 vs. 31 vs. 33 U/L), aspartate aminotransferase (median, 24 vs. 29 vs. 30 U/L), γ-glutamyl transferase (median, 22 vs. 33 vs. 43 U/L), alkaline phosphatase(median, 59 vs. 64 vs. 65 U/L), total bilirubin (median, 9.8 vs. 10.8 vs. 11.2 μmol/L), direct bilirubin (median, 3.5 vs. 3.9 vs. 4.2 μmol/L), lactate dehydrogenase (median, 252 vs. 268 vs. 286 U/L) and creatine kinase-MB (median, 0.89 vs. 0.96 vs. 1.12 ng/mL) were increased, although no significant differences were observed between the normal and obesity groups in aspartate aminotransferase, alkaline phosphatase, total bilirubin, direct bilirubin and lactate dehydrogenase. These results indicated that the COVID-19 patients with obesity may have more severe organ dysfunction or injury at admission** (Table 2).**

Compared to the normal group, the obesity group had higher levels of CD19+ cell counts (median, 129 vs. 195 /μL) and CD19+ cell percentage (14.7 vs. 19.1 %) and lower levels of CD3+ cell percentage (68.8% vs. 62.4%). In addition, the CD4+ cell and CD8+ cell counts were lower in the obesity group, although no significant differences were detected; the limited number of patients in the obesity group may be responsible for this finding. Therefore, a more severe dysfunction of lymphocyte immunity may be present in patients in the obesity group **(Table 2).**

### Manifestations of chest CT at admission and treatment during hospitalization in COVID-19 patients

Regarding lung CT findings at admission, we found that no significant differences were detected among three groups in several manifestations, including unilateral pneumonia, bilateral pneumonia, ground-glass opacity, reticular/linear and air bronchogram. Compared to the normal group, the overweight group had more patients with consolidation shadows in lung CT **(Table 3).**

Regarding treatment, there were no differences among the three groups in major treatment regimens, such as antiviral therapy, antibacterial therapy, antifungal therapy, glucocorticoids, immunoglobulin, traditional Chinese medicine and oxygen therapy **(Table 4).**

### The association between overweight or obesity and severity, organ injury or designed endpoint

Compared to the normal weight group, the proportion of mild-moderate patients (42.2 vs. 19.0 %) was decreased in the obesity group. The proportions of sev**e**re+critical illness (47.8 vs. 81.0 %) were higher in the obesity group than those in the normal weight group. No significant differences were observed between the normal and overweight groups. Compared to the overweight group, the obesity group had a higher proportion of patients with complications (11.7 vs. 23.8 %), while, no significant differences were observed between normal group and obesity group. The designed endpoint was a composite of mechanical ventilation or ICU admission. After every patient was followed for 30 days, the differences were not significant among three groups in the endpoint **(Table 4).**

After adjusting for age, sex, comorbidity (e.g., hypertension, diabetes, coronary artery disease, arrhythmia, cerebrovascular disease, COPD, asthma, chronic renal disease and chronic liver disease), multivariate logistic regression analysis revealed that COVID-19 patients with obesity were more likely to develop severe+critical illness (Odd ratio 3.586, 95% CI 1.550-8.298, P=0.003). However, the association between obesity and the designed endpoint, critical illness or organ damage was not significant. In addition, overweight was not found to be significantly associated with critical illness, severe+critical illness, organ damage and primary endpoint** (Table 5).**

### Depletion of CD4+ cells may contribute to developing severe illness in the subgroup of COVID-19 patients with obesity

**Figure 2:**
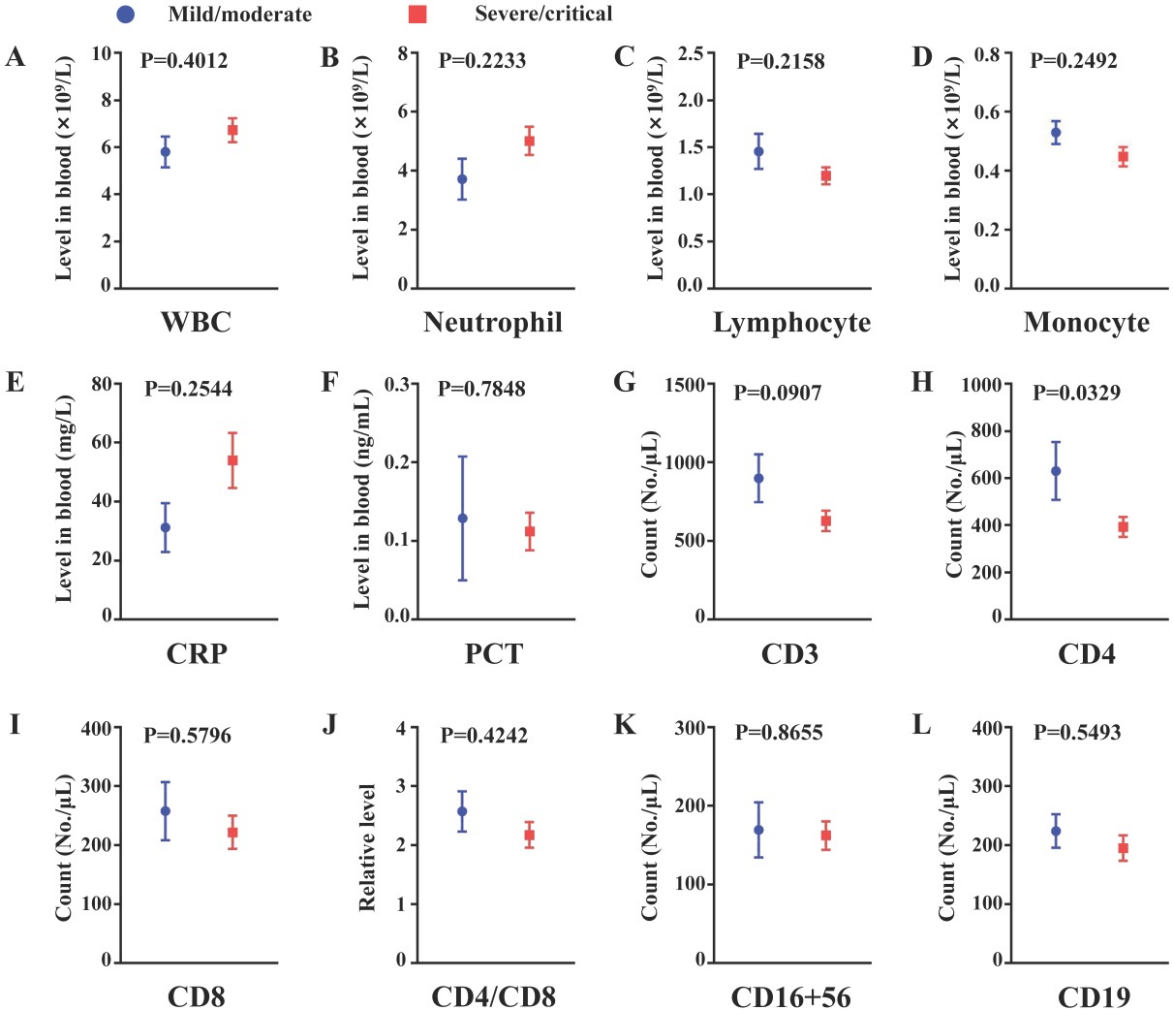
The comparison of inflammation and cellular immunity between non-severe and severe patients from obesity subgroup. (A) White blood cell count (B) Neutrophil count; (C) Lymphocyte count; (D) Monocyte count; (E) C-reactive protein level in peripheral blood; (F) Procalcitonin level in peripheral blood; (G) CD3+ cell count; (H) CD4+ cell count; (I) CD8+ cell count; (J) Relative CD4/CD8 level; (K) CD16+56+ cell count; (L) CD19+ cell count. n=8 in the mild/moderate group, n=27-34 in the severe/critical group. Data was presented by mean±SEM and compared using student t' test, p<0.05 was considered significantly different.

In the subgroup of COVID-19 patients with obesity, compared to the nonsevere group, the levels of CD4 were significantly decreased in the severe group. Similar decreased trends in CD3+ cells, CD8+ cells, CD4+/CD8+ ratio, CD19+ cells and lymphocytes and, in turn, increased trends in WBC, neutrophils and CRP were observed, while the differences were not significant. If the size of the obesity group was increased, these trends may be significant. The results indicated that depletion of CD4+ T cells may be responsible for the development of severe illness in the subgroup of COVID-19 patients with obesity **(Figure 2A-L).**

## Discussion

Our present study found that obese patients had an increased risk of developing severity in patients with COVID-19. Obese patients were more likely to have hypertension, as well as severe secondary infection, metabolic disorder, organ dysfunction, and disorder of cellular immunity.

Obesity is another global health crisis. In 2012, the prevalence of obesity in adults was 11.9% in China and has increased rapidly from 2002 to 2012 17. In 2013, the proportions of adults with BMI ≥ 25.0 kg/m^2^ have increased to 36.9% in adult men and 38% in women, worldwide 18. During the COVID-19 pandemic, obesity has become one of the most prevalent comorbidities, especially in the USA 5. Obesity can cause damage to expiratory reserve volume, functional capacity and respiratory system compliance, which may be associated with the aggravation of disease 19. In the epidemic of HIN1 influenza virus, several studies analyzing the effect of obesity revealed that obesity is strongly associated with severe illness 20-22. Additionally, there was a large fraction of obese patients among the patients admitted into the ICU 20, 21. In our study, we found that obesity was associated with an increased risk of developing severity in patients with COVID-19. The conclusion was consistent with the reports from Cai et al. 23 in Shenzhen, China. In addition, Haywood et al. 24 concluded that obesity was a risk factor for hospital admission, suggesting that obese patients needed more hospital care. In our study, we found that obesity did not have an obvious effect on the development of endpoint or organ damage. In a cohort of COVID-19 patients from New York, Palaiodimos et al. 25 indicated that severe obesity (BMI > 35.0 kg/m^2^) was independently associated with higher in-hospital mortality. In another prospective observational cohort study 26, obesity was also associated with higher in-hospital mortality. The lack of BMI data for patients who died and the limited size of the obesity group may be responsible for the negative results. In addition, the proportion of severe obesity was low in our study, which may be due to the relatively low rate of severe obesity in China 27. Since severe patients with COVID-19 are associated with poor prognosis, we indicated that COVID-19 patients with obesity may be more likely to develop poor outcomes. Obese patients with COVID-19 may need more attention during hospitalization, even in countries with low obesity rates.

Obese patients are more likely to have diabetes mellitus, hypertension and other cardiovascular diseases 6-8. Comorbidities including hypertension and diabetes mellitus are associated with mortality in patients with COVID-19 10, 28, 29. In addition, complications including shock, acute heart injury, kidney injury and liver injury are commonly observed in severe or critical patients with COVID-19 and can also increase the risk of developing severe illness and poor outcomes 30,31. In our study, the obesity group had a higher proportion of patients with preexisting diseases; in particular, patients with hypertension. In addition, organ dysfunction was more serious in the obesity group than that in the normal weight group at admission. These characteristics may be responsible for the higher proportions of severe+critical COVID-19 in the obesity group. Moreover, obese patients with COVID-19 were more likely to have secondary bacterial infection and relatively high levels of CRP, which also contributed to the development of critical illness. A previous study has indicated that chronic inflammation was present in obese patients 32. The co-existing inflammation may have aggravated the secondary infection in obese patients with COVID-19.

Obese patients with COVID-19 had higher levels of fasting blood glucose, triacylglycerol and uric acid and lower levels of high-density lipoprotein, indicating that obese patients with COVID-19 were more likely to have metabolic disorder. Costa et al. 33 has suggested that metabolic disorder may be a risk factor for affecting the progression and prognosis of COVID-2019.

Changes in peripheral lymphocyte subsets have been observed in COVID-19 patients 34. SARS-CoV-2 infection decreased the peripheral levels of CD3+ cells, CD4+ cells and CD8+ cells, which were further reduced in severe illness 35, 36. Wang et al. 36 also found that the levels of CD19+ cells, not CD16+56+ cells, were lower in severe cases of COVID-19 than those in mild cases. In our study, we found that percentages of CD3+ cells were lower in the obesity group than those in the normal weight group. The counts of CD4+ cells and CD8+ cells were also decreased in the obesity group, while the differences were not significant. The reductions of these cells may contribute to the high percentages of severe and critical COVID-19 in obese patients. In addition, we showed that the levels of CD19+ B cells were higher in obese patients. Although B cells help the body to generate antibody, they can also promote inflammation in obesity 37. B cells have been recognized as having an important effect on the control of hepatitis B virus 38. Therefore, more severe adaptive immune disorders may exist in obese patients with COVID-19, and the detailed roles of CD19+ B cells will require further studies in the future. In the obesity subgroup, compared to the nonsevere patients, peripheral levels of CD4+ cells were decreased in severe COVID-19 patients. The results suggested that depletion of CD4+ cells was associated with the increased risk of progression of COVID-19 in the obesity subgroup.

### Limitations

First, the BMI data were obtained from the follow-up of survival patients and the follow-up time was two weeks after discharge. This gave rise to several potential concerns: the accuracy of the BMI data measurement could be questionable; the BMI was not measured upon the COVID-19 diagnosis but rather was collected at 14-day follow-up, BMI at this point might not be the same as the BMI during in-hospital period, therefore, the BMI data might be either underestimated or overestimated, especially for those whose BMI was at the borderline; some of the most critical patients who did not survive from COVID-19 were not included in this study. Second, the size of the obesity group in our study was quite small. Third, only a small number of patients had data regarding interleukins, interferon γ, and other cytokines, all of which would help to analyze the role of the inflammatory storm in obese patients with COVID-19.

## Conclusion

Obese patients with COVID-19 were more likely to have hypertension, secondary infection, organ dysfunction, metabolic disorders and dysfunction of lymphocyte immunity. Obesity was associated with an increased risk of developing severe/critical illness in patients with COVID-19.

## Figures and Tables

**Table 1:** Basic characteristics of patients with COVID-19 among normal, overweight and obesity group

Characteristics	Normal (n=242)	Overweight(n=179)	Obesity (n=42)
**Age, years**	62 (49, 68)	59 (50, 67)	63 (45, 68)
**Male**	110 (45.5)	91 (50.8)	23 (54.8)
**Initial symptom**			
Fever	198 (81.8)	149 (83.2)	31 (73.8)
Cough	143 (59.1)	110 (61.5)	27 (64.3)
Chest distress	50 (20.7)	45 (25.1)	7 (16.7)
Dyspnea	53 (21.9)	60 (33.5)*	8 (19.0)
Fatigue	80 (33.1)	60 (33.5)	12 (28.6)
Muscle ache	19 (7.9)	11 (6.1)	3 (7.1)
Anorexia	23 (9.5)	19 (10.6)	8 (19.0)
Diarrhea	31 (12.8)	20 (11.2)	6 (14.3)
**Signs at admission**			
Temperature *^A^*, ℃	36.7 (36.5, 37.3)	36.7 (36.4, 37.3)	36.8 (36.5, 37.0)
Heart rate *^B^*, bpm	83 (78, 96)	85 (78, 94)	90 (81, 102)*
Respiratory rate *^B^*, bpm	20 (18, 20)	20 (18, 20)	20 (19, 21)
Finger oxygen saturation *^C^*, %	98 (96, 99)	96 (95, 98)	96 (95, 97.75)*
**Comorbidity (n, %)**			
Hypertension	52 (21.5)	56 (31.3)*	18 (42.9)*
Diabetes mellitus	25 (10.3)	22 (12.3)	4 (9.5)
Coronary artery disease	12 (5.0)	11 (6.1)	4 (9.5)
Arrythmia	8 (3.3)	2 (1.1)	1 (2.4)
COPD/Asthma	11 (4.5)	3 (1.7)	0
Cerebrovascular disease	5 (2.1)	2 (1.1)	2 (4.5)
Chronic kidney disease	6 (2.5)	2 (1.1)	0
Chronic liver disease	7 (2.9)	7 (3.9)	1 (2.4)
Only one comorbidity	55 (22.7)	52 (29.1)	12 (28.6)
≥ 2 comorbidities	32 (13.2)	25 (14.0)	10 (23.8)
**Duration before admission**	10 (7, 13)	11 (8, 15)	11 (8,14)

Values are number (percentage) or Medium (IQR) unless stated otherwise. Continuous variables were compared by the Mann-Whitney test and categorical variables were compared by chi-square tests, or fisher's exact test. * p<0.05 vs. Normal weight group; # p<0.05 vs. Overweight group. Abbreviation: COPD, chronic obstructive pulmonary disease. The total number of patients with available data in different groups (n1, n2 and n3 indicates the number in normal weight, overweight and obesity group, respectively): ***A***: n1=241, n2=178, n3=38; ***B***: n1=239, n2=178, n3=39; ***C***: n1=153, n2=137, n3=30.

**Table 2:** First laboratory findings of COVID-19 patients within 3 days of admission among normal, overweight and obesity group

	Normal (n=242)	Overweight (n=179)	Obesity (n=42)
**Blood routine *^A^***			
White blood cell count, ×10^9^/L	4.88 (3.81, 6.49)	5.76 (4.42, 7.2)*	6.49 (4.27, 7.79)*
Neutrophil percentage, %	66.1 (56.2, 75.8)	68.7 (60.9, 79.3)*	71.7 (62.4, 79.0)
Neutrophil count, ×10^9^/L	3.23 (2.31, 4.63)	3.91 (2.7, 5.38)*	4.34 (2.74, 6.32)*
Lymphocyte percentage, %	23.3 (15.4, 31.8)	19.6 (12.3, 28.3)*	20.2 (13.4, 28.7)
Lymphocyte count, ×109/L	1.08 (0.81, 1.46)	1.12 (0.79, 1.53)	1.12 (0.89, 1.65)
Monocyte percentage, %	8.6 (6.3, 10.9)	8.5 (6.1, 10.4)	7.6 (5.3, 8.7)*
Monocyte count, ×109/L	0.42 (0.31, 0.56)	0.45 (0.35, 0.61)	0.45 (0.34, 0.57)
Basophil count, ×109/L	0.01 (0.01, 0.02)	0.01 (0.01, 0.03)*	0.02 (0.01, 0.03)*
Eosinophil count, ×109/L	0.01 (0, 0.06)	0.03 (0, 0.08)	0.01 (0, 0.09)
Blood platelet count, ×109/L	214 (160, 273)	230 (185, 286)*	230 (168, 308)
**Inflammation**			
C-reactive protein ***^B^***, mg/L	33.1 (6.35, 70.15)	27 (6.65, 60.85)	41.65 (9.28, 70.5)
hsC-reactive protein ***^C^***, mg/L	5 (5, 5)	5 (5, 5)	5 (5, 5)
Procalcitonin ***^D^***, ng/mL	0.046 (0.028, 0.096)	0.051 (0.036, 0.082)	0.06 (0.04, 0.08)
**Intravenous glucose *^E^*, mmol/L**	5.33 (4.79, 6.61)	5.76 (5.1, 7.79)*	6.29 (5.13, 7.15)
**Blood lipid *^F^***			
Total cholesterol, mmol/L	3.8 (3.26, 4.47)	3.75 (3.33, 4.43)	3.85 (3.33, 4.40)
Triglyceride, mmol/L	1.14 (0.9, 1.52)	1.19 (0.97, 1.68)	1.34 (1.09, 2.16)*
Low density lipoprotein, mmol/L	2.27 (1.85, 2.91)	2.45 (1.94, 2.88)	2.46 (2.01, 2.97)
High density lipoprotein, mmol/L	0.97 (0.81, 1.14)	0.87 (0.77, 1.05)*	0.88 (0.72, 1.01)*
**Blood electrolyte *^F^***			
K+	3.9 (3.6, 4.4)	3.9(3.6, 4.2)	4.0 (3.6, 4.3)
Na+	141 (138, 144)	141 (138, 144)	141 (137, 144)
**Liver function**			
Alanine aminotransferase ***^G^***, U/L	20 (15, 32)	31 (20, 54)*	33 (21, 47)*
Aspartate aminotransferase ***^G^***, U/L	24 (19, 34)	29 (21, 43)*	30 (22, 42)
γ-glutamyltransferase ***^H^***, U/L	22 (15, 35)	33 (22, 57)*	43 (25, 64)*
Alkaline phosphatase ***^H^***, U/L	59 (49, 70)	64 (54, 79)*	65 (54, 84)
Total bilirubin ***^H^***, μmol/L	9.8 (7.6, 12.6)	10.8(8.1, 14.2)*	11.2(8.8, 14.8)
Direct bilirubin ***^H^***, μmol/L	3.5 (2.5, 4.8)	3.9 (2.9, 5)*	4.2 (2.9, 5.4)
**Kidney Function *^I^***			
Creatinine, μmol/L	60 (49, 72)	61 (50, 72)	65 (50, 76)
Blood urea nitrogen, mmol/L	4.29 (3.41, 5.59)	4.4 (3.58, 5.59)	4.5 (3.95, 5.72)
Blood uric acid, μmol/L	232 (187, 298)	253 (211, 319)*	305 (231, 357)*#
eGFR, mL/min	98.4 (90.0, 106.9)	98.9 (92.0, 108.3)	97.3 (85.6, 112.1)
**Myocardial injury**			
Creatine kinase ***^J^***, ng/mL	57 (37, 87)	59 (39, 106)	64 (42, 115)
Creatine kinase-MB ***^K^***, ng/mL	0.89 (0.54, 1.35)	0.96 (0.66, 1.26)	1.12 (0.76, 2.2)*
Lactate dehydrogenase ***^L^***, U/L	252 (203, 310)	268 (217, 340)*	286 (208, 356)
Myoglobin ***^M^***, g/L	36.54 (25.26, 58.8)	40.03 (27.92, 61.3)	45.04 (30.57, 80.48)
Hypersensitive troponin I ***^N^***, ng/mL	0.006 (0.006, 0.009)	0.006 (0.006, 0.008)	0.006 (0.006, 0.009)
NTpro-BNP ***^O^***, pg/ml	114.7 (47.3, 235.2)	105.1 (34.6, 235.3)	152.7 (61.5, 370.1)
**Coagulation function *^P^***			
D-Dimer, mg/L	0.62 (0.36, 1.44)	0.63 (0.38, 1.36)	0.72 (0.4, 2.67)
Prothrombin time activity, %	84.7 (75.9, 95)	84.7 (77.3, 93.3)	85.4 (82.2, 91.4)
APTT, sec	28.3 (26.3, 31.3)	27.8 (25.6, 30.2)*	27.7 (25.5, 31.2)
International normalized ratio	1.03 (0.97, 1.08)	1.03 (0.97, 1.07)	1.03 (0.99, 1.05)
Thrombin time, sec	17.6 (16.7, 18.4)	17.6 (16.6, 18.5)	17.4 (16.8, 18.2)
Prothrombin time, sec	12 (11.4, 12.6)	11.95 (11.425, 12.5)	12 (11.6, 12.2)
Antithrombin III activity, %	89.9 (82.2,97.8)	88.8 (81.6, 97.8)	87.6 (82.1, 95.9)
Fibrin degradation products, mg/L	2.28 (0.94, 4.99)	1.93 (0.98, 5.38)	2.8 (1.81, 7.35)
Fibrinogen, g/L	4.56 (3.42, 5.46)	4.56 (3.73, 5.7)	4.69 (3.52, 5.87)
**Cellular immunity *^Q^***			
CD16+56+ cell percentage, %	13.1 (8.3, 21.5)	12.5 (8.5, 18.8)	16.4 (10.1, 21.3)
CD16+56+ cell counts, /μL	119 (71, 180)	117 (77, 175)	159 (104, 193)
CD19+ cell percentage, %	14.7 (11.0, 19.3)	17.5 (13.2, 21.6)*	19.1(15.2, 21.3)*
CD19+ cell counts, /μL	129 (9, 184)	159 (106, 247)*	195 (124, 280)*
CD3+ cell percentage, %	68.8 (58.1, 75.6)	67.2 (58.0, 71.9)	62.4 (53.4, 69.5)*
CD3+ cell counts, /μL	623 (427, 882)	659 (441, 927)	628 (424, 858)
CD4+ cell percentage, %	39.4 (32.0, 46.5)	40.4 (33.8, 46.1)	39.7 (33.4, 45.5)
CD4 cell counts, /μL	375 (249, 513)	420 (251, 576)	359 (241, 535)
CD8+ cell percentage, %	23.8 (17.2, 30.1)	21.5 (16.5, 28.0)	18.3 (14.0, 27.2)
CD8+ cell counts, /μL	231 (128, 335)	207 (132, 315)	212 (114, 321)
CD4/CD8 ratio	1.67 (1.21, 2.41)	1.8 (1.32, 2.7)	2.14 (1.38, 3.17)
**Arterial blood gas analysis *^R^***			
PH value	7.42 (7.39, 7.46)	7.43 (7.37, 7.46)	7.44 (7.38, 7.46)
Partial pressure of oxygen, mmHg	88 (70, 112)	83 (70, 109)	78 (55, 87)
Arterial oxygen saturation, %	97 (94, 98)	96 (94, 98)	95 (90, 97)
Arterial lactate acid, mmol/L	1.9 (1.5, 2.5)	1.9 (1.4, 2.6)	2.2 (1.6, 2.6)

Values are Medium (IQR) unless stated otherwise. Continuous variables were compared by the Mann-Whitney test. * p<0.05 vs. Normal weight group; # p<0.05 vs. Overweight group. Abbreviation: hsC-reactive protein, high-sensitive reactive protein; eGFR, estimated glomerular filtration rate; NTpro-BNP, N-terminal pro-Brain Natriuretic Peptide; APTT, activated partial thromboplastin time. The total number of patients with available data in different groups (n1, n2 and n3 indicates the number in normal weight, overweight and obesity group, respectively): ***A***: n1=237, n2=175, n3=42; ***B***: n1=199, n2=155, n3=36; ***C***: n1=202, n2=159, n3=36; ***D***: n1=218, n2=170, n3=39; ***E***: n1=238, n2=179, n3=42; ***F***: n1=230, n2=173, n3=42; ***F***: n1=238, n2=178, n3=41; ***G***: n1=238, n2=179, n3=42; ***H***: n1=232, n2=176, n3=42; ***I***: n1=238, n2=179, n3=42; ***J***: n1=232, n2=173, n3=41; ***K***: n1=200, n2=140, n3=37; ***L***: n1=232, n2=171, n3=41; ***M***: n1=199, n2=140, n3=36; ***N***: n1=201, n2=145, n3=36; ***O***: n1=175, n2=130, n3=31; ***P***: n1=215, n2=161, n3=39; ***Q***: n1=198, n2=144, n3=33; ***R***: n1=105, n2=82, n3=17.

**Table 3:** Lung CT findings of COVID-19 patients at admission among normal, overweight and obesity group

Features of lung CT	Normal (n=129)	Overweight (n=86)	Obesity (n=18)
**Pneumonia**	129 (100)	85 (98.8)	18 (100)
Unilateral lung	13 (10.1)	2 (2.3)	0
Bilateral lung	116 (89.9)	83 (96.5)	18 (100)
Ground-glass opacity	104 (80.6)	71 (82.6)	14 (77.8)
reticular/linear	35 (27.1)	25 (29.1)	5 (27.8)
Air bronchogram	15 (11.6)	11 (12.8)	4 (22.2)
Consolidation shadow	15 (11.6)	22 (25.6)*	5 (27.8)

Values are number (percentage). Categorical variables were compared by chi-square tests, or fisher's exact test. * p<0.05 vs. Normal weight group; # p<0.05 vs. Overweight group. Abbreviation: CT, computerized tomography.

**Table 4:** Treatment, typing, complication and endpoint of COVID-19 patients among normal, overweight and obesity group

	Normal (n=242)	Overweight (n=179)	Obesity (n=42)
**Treatment**			
Antiviral therapy	237 (97.9)	170 (95.0)	41 (97.6)
Antibacterial therapy	200 (82.6)	141 (78.8)	37 (88.1)
Antifungal therapy	5 (2.7)	2 (1.1)	1 (2.4)
Glucocorticoid therapy	106 (43.8)	72 (40.2)	20 (47.6)
Immunoglobulin therapy	129 (53.3)	79 (44.1)	25 (59.5)
Traditional Chinese medicine	187 (77.3)	128 (71.5)	33 (78.6)
Oxygen therapy	213 (88.0)	161 (89.9)	37 (88.1)
**Typing**			
Mild-moderate	102 (42.2)	61 (34.1)	8 (19.0)*
Severe	119 (49.2)	99 (55.3)	27 (64.3)
Critical	21 (8.7)	19 (10.6)	7 (16.7)
**Complication**	32 (13.2)	21 (11.7)	10 (23.8)#
Shock	4 (1.7)	3 (1.7)	2 (4.8)
Acute myocardial injury	16 (6.6)	8 (4.5)	4 (9.5)
Acute kidney injury	1 (0.4)	2 (1.1)	0
Acute liver injury	15 (6.2)	10 (5.6)	4 (9.5)
**Endpoint**	21 (8.68)	19 (10.6)	5 (11.9)

Values are number (percentage). Categorical variables were compared by chi-square tests, or fisher's exact test. * p<0.05 vs. Normal weight group; # p<0.05 vs. Overweight group. Endpoint was a composite of mechanical ventilation or admission of intensive care unit, after all patients were followed for 30 days.

**Table 5:** The association between obesity or overweight and severity, organ damage, or endpoint in patients with COVID-19

	Odd Ratio	95% CI	P value
**Critical illness**			
**Model 1**			
Normal	-	-	-
Overweight	1.250	0.650-2.401	0.504
Obesity	2.105	0.833-5.317	0.116
**Model 2**			
Overweight	1.240	0.629-2.445	0.534
Obesity	1.973	0.744-5.231	0.172
**Severe + Critical illness**			
**Model 1**			
Overweight	1.409	0.944-2.104	0.093
Obesity	3.096	1.376-6.970	0.006*
**Model 2**			
Overweight	1.443	0.953-2.185	0.083
Obesity	3.586	1.550-8.298	0.003*
**Organ damage**			
**Model 1**			
Overweight	0.872	0.485-1.570	0.649
Obesity	2.051	0.920-4.571	0.079
**Model 2**			
Overweight	0.894	0.490-1.634	0.716
Obesity	2.005	0.875-4.593	0.100
**Endpoint**			
**Model 1**			
Overweight	1.250	0.650-2.401	0.504
Obesity	1.422	0.505-4.006	0.505
**Model 2**			
Overweight	1.231	0.622-2.434	0.551
Obesity	1.299	0.440-3.838	0.636

Endpoint was a composite of mechanical ventilation or admission of intensive care unit, after all patients were followed for 30 days. Model 1: Univariate regression analysis. Model 2: Multivariate regression analysis with adjusting for sex, age and comorbidities (e.g. hypertension, diabetes, coronary artery disease, arrhythmia, cerebrovascular disease, COPD, asthma, chronic renal disease and chronic liver disease).
